# [Corrigendum] P2Y2 receptor promotes the migration and invasion of breast cancer cells via EMT‑related genes Snail and E‑cadherin

**DOI:** 10.3892/or.2024.8770

**Published:** 2024-07-03

**Authors:** Ying Qiu, Yan Liu, Wei-Hua Li, Hong-Quan Zhang, Xin-Xia Tian, Wei-Gang Fang

Oncol Rep 39: 138–150, 2018; DOI: 10.3892/or.2017.6081

Subsequently to the publication of the above paper, an interested reader drew to the authors' attention that there appeared to be two instances of overlapping data panels comparing between the cell migration and invasion assay data shown in Figs. 4 and 6 on p. 143 and 145, respectively, such that data which were intended to represent the results from differently performed experiments had apparently been derived from the same original sources. In addition, the authors themselves realized that incorrect western blotting data for Snail protein in Fig. 10A on p. 147 had been included in the figure.

The authors were able to re-examine their original data files, and realized that the affected data panels in these figures had inadvertently been incorporated into them incorrectly. The revised versions of Figs. 4, 6, and 10, featuring the correct data for the ‘NC / Control’ panels in Fig. 4B and C and the ‘siRNA2 / ATP 12 h’ panels in Fig. 4A and B, a replacement data panel for the ‘siRNA1 / Control’ experiment in Fig. 6, and the correct western blotting data for Snail protein in Fig. 10A (together with a revised histogram for the MCF7 cell line relating to Fig. 10A) are shown on the next three pages. The authors wish to emphasize that the errors made in compiling these figures did not affect the overall conclusions reported in the paper, and they are grateful to the Editor of *Oncology Reports* for allowing them the opportunity to publish this corrigendum. All the authors agree to the publication of this corrigendum, and also apologize to the readership for any inconvenience caused.

## Figures and Tables

**Figure 4.**
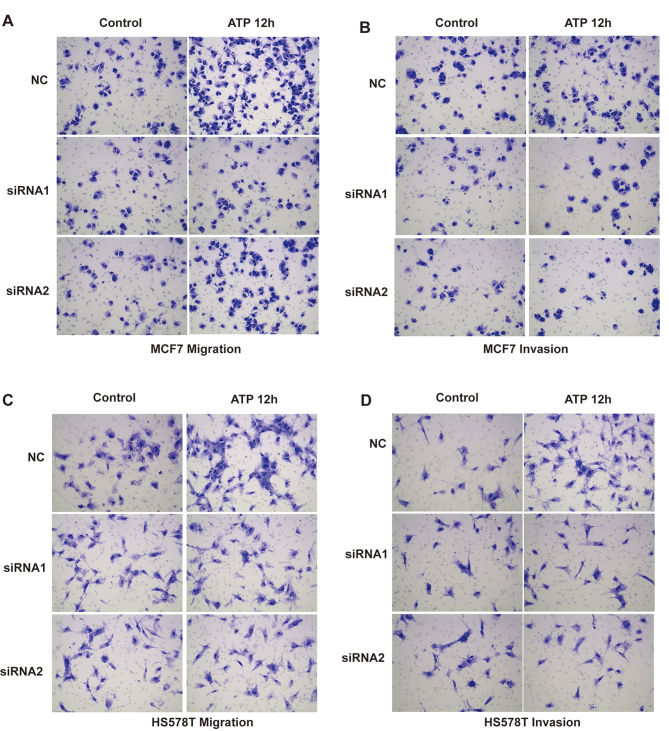
Effects of P2Y2 receptor knockdown on ATP-mediated migration and invasion in breast cancer cells. (A-D) *In vitro* migration and invasion assays were carried out as described in Materials and methods in the absence (control) or presence of 100 µM ATP (ATP 12 h). At least 3 independent experiments were performed.

**Figure 6.**
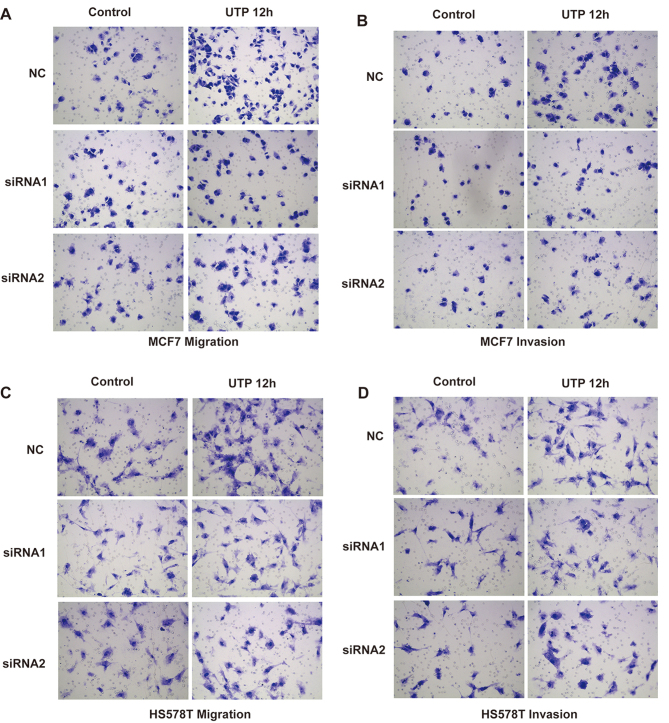
Effects of P2Y2 receptor knockdown on UTP-mediated migration and invasion in breast cancer cells. (A-D) In vitro migration and invasion assays were carried out as described in Materials and methods in the absence (control) or presence of 100 µM UTP (UTP 12 h). At least 3 independent experiments were performed.

**Figure 10.**
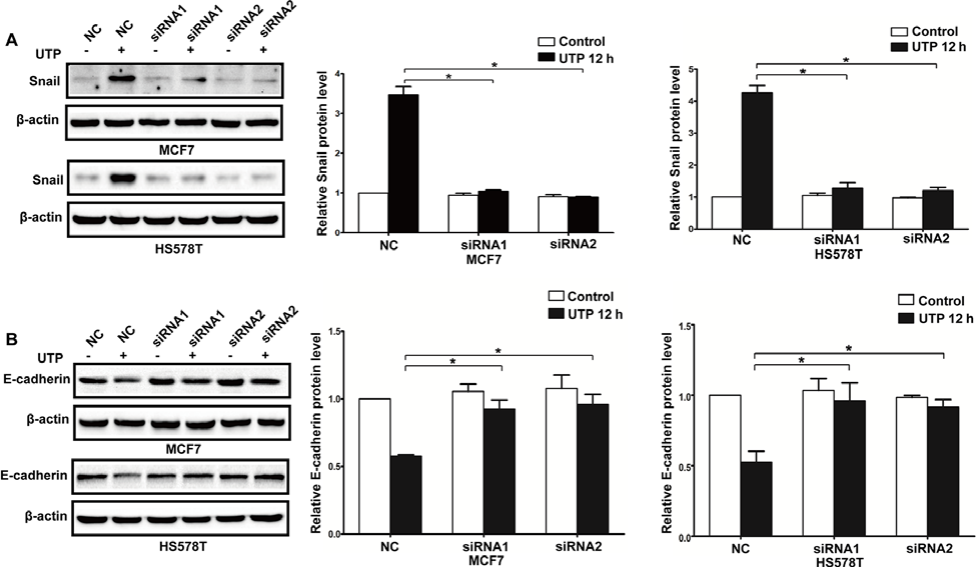
Knockdown of the P2Y2 receptor attenuates UTP-induced expression changes in EMT-related genes in breast cancer cells. P2Y2-silenced cells (siRNA1 and siRNA2) and control siRNA cells (NC) were treated with or without 100 µM UTP for 12 h (UTP 12 h). Western blot experiments were performed to examine protein levels of (A) Snail and (B) E-cadherin. Expression levels of these proteins were normalized to their respective expression in the control cells (without UTP). Data are presented as mean ± SD (vertical bars). At least 3 independent experiments were performed; *P<0.05.

